# Measuring the effectiveness of a car seat program in an urban, level one pediatric trauma center

**DOI:** 10.1186/s40621-021-00313-1

**Published:** 2021-09-13

**Authors:** Ross Budziszewski, Rochelle Thompson, Thomas Lucido, Janelle Walker, Loreen K. Meyer, L. Grier Arthur, Harsh Grewal

**Affiliations:** 1 St. Christopher’s Hospital for Children, Philadelphia, PA USA; 2 Drexel University College of Medicine, Philadelphia, PA USA

**Keywords:** Car seat safety, Hospital outreach, Prevention programming

## Abstract

**Background:**

Motor vehicle collisions (MVCs) are a significant safety issue in the United States. Young children are disproportionally impacted by car accidents and suffer high rates of injuries and mortality. When used properly, car seats have been found to reduce the severity of injuries. However, individuals from low-income areas often do not have access to education or car seats compared to those in suburban or higher income areas. Therefore, the goal of the present study was to measure the effectiveness of a car seat program in an urban, Level I Pediatric Trauma Center on caregiver car seat knowledge.

**Methods:**

Caregivers (*N* = 200) attended a single, one-hour car seat educational program with a Child Passenger Safety Technician (CPST). The sessions included educational and hands-on components, where caregivers were asked to complete a seven-item pre-post knowledge assessment. For completion of the course, caregivers received a car seat for their child.

**Results:**

A paired *t*-test revealed that the workshop significantly increased caregiver knowledge from pre-post: *t* (199) = − 12.56, *p* < .001; *d* = 1.27. McNemar’s Chi-Square analyses displayed that caregivers increased in all knowledge categories (*p* < .001).

**Conclusions:**

While caregivers in urban areas or in low-income areas may have less access to resources, hospital-led car seat courses can increase knowledge of proper car seat usage in these communities. These findings should be used to establish programs in hospitals in areas where these resources are not readily available to caregivers.

## Background

Motor vehicle collisions (MVCs) are a significant safety issue in the United States. The Centers for Disease Control and Prevention (CDC) reported that approximately 3,000,000 non-fatal injuries were related to MVCs in 2019 (The Centers for Disease Control and Prevention 2020). As of 2019, MVC related injuries resulted in roughly 700,000 hospitalizations and 6000 deaths (National Highway Traffic Safety Administration 2020). Young children are disproportionately impacted by MVCs compared to the general population. MVCs are the leading cause of death in children under the age of 12, with almost 700 deaths and over 160,000 injuries annually (The Centers for Disease Control and Prevention 2020).

Over the past two decades, there has been a substantial decline in reported child fatalities from MVCs (Lee et al. 2018; Singh et al. 2016). The decline in child fatalities can be credited to increasing passenger safety technology, developing legislation to keep children safe, and increasing safety interventions (Ma et al. 2013; Pressley et al. 2009). Notably, implementing laws that require the use of child car restraints has decreased injuries and deaths related to MVCs (Henary et al. 2007). While legislation has made car seats required, caregivers must still possess knowledge of proper installation and guidelines to ensure that their child is safe.

As such, car seat education interventions have been successful at increasing caregiver and provider knowledge of proper car seat usage (Tessier 2010). Specifically, car seat interventions may provide caregivers important information such how to choose the appropriate type of seat, understanding current guidelines and laws, and requirements to help ensure maximal protection against the forces of a crash (Tessier 2010; Macy and Freed 2012). The CDC (2017) recommends that parents use a rear-facing seat until 2 years of age, a forward-facing seat until age five or when a child is over 40 pounds, and a booster seat until a car’s normal seatbelt can fit properly (Rice and Anderson 2009). Installation of car seats is extremely important, but it is imperative that parents understand how to tighten straps, height and weight requirements, what clothing their child should be wearing while in the car seat, and child behaviors that may contribute to determining the most appropriate car seat for the child (The Center for Disease Control and Prevention 2020).

Given the importance of children being fitted properly in car seats, all caregivers should have access to resources to help them understand appropriate use. However, this is not always the case, especially for caregivers who reside in areas of lower socioeconomic statuses (SES) or urban areas (Tessier 2010). This disparity is displayed through the decreases in death and injury among individuals of higher SES over the past two decades, while those in lower SES have seen a slower decline (Sauber-Schatz et al. 2014). Further, caregiver behavior is different based on SES, with individuals from low-income areas reporting significantly less car seat usage than those in higher income areas (Harper et al. 2015; Laflamme et al. 2010). Some of this difference can be attributed to low-income families having less access to health and preventative resources as well as less financial ability to obtain often expensive items such as car seats for their children (Rok Simon et al. 2016; Fleary et al. 2013).

One potential way to help address some of the disparities in car seat use is for hospitals and facilities (e.g., external organizations such as YMCAs or local resource centers) in low-income areas to offer community outreach efforts aimed at educating caregivers on how to properly install car seats and provide information on legislative guidelines. These outreach efforts can be led by Child Passenger Safety Technicians (CPSTs) who are trained to work in community settings to help educate parents on car passenger safety (Ross et al. 2007). Previous literature has shown the efficacy of CPST led programs on increasing caregiver car seat knowledge, yet the impact that at an educational program can have in a low-income, clinical setting has not been examined (Louis and Louis 1997; Schwebel et al. 2017). Therefore, the goal of the present study was to measure the effectiveness of a car seat program in an urban, Level I Pediatric Trauma Center on caregiver car seat knowledge to promote child safety.

## Methods

### Program setting

The course was held at an urban, Level I Pediatric Trauma Center in the Northeast United States. The hospital itself is a 188-bed unit and it houses one of the busiest level-one pediatric emergency departments in the United States with over 70,000 annual visits (Tessier 2010). The hospital is located in the poorest congressional district per capita in its state and over 80% of patients use a form of Medicaid (Brubaker 2020). The hospital has a strong outreach program with two CPSTs to assist with car seat education and installation.

### Program description and measures

The current study was acknowledged and accepted by the authors’ Institutional Review Board (IRB). There program posed no immediate risk to caregivers who were included. Identifying information such as names, race, and contact information was not collected to protect participant privacy. CPSTs (*N* = 2) facilitated the program’s sessions. A total of 20, one-hour sessions were completed, with roughly ten caregivers per session. Caregivers were identified by physicians, social work, local outside resource centers, or the hospital’s injury prevention coordinator. The program took place at the hospital’s educational conference room and occurred approximately once a month, or when enough caregivers were referred to warrant a session. In addition, when caregivers were already at the hospital with their child(ren), the hospital’s injury prevention coordinator provided an individualized course as needed, although this was relatively uncommon. The course was organized in four parts: (1) an initial knowledge pre-test, (2) a didactic lecture with demonstration of how to use a car seat, (3) a hands-on test of car seat usage, and (4) a knowledge post-test. Caregivers received an appropriate seat based on the child’s weight and height their child and assistance from a CPST to properly install the seat in their car.

The lecture material consisted of information on car seat laws, factors contributing to choosing the appropriate car seat, age recommendations, clothing requirements in car seats, harness location and tightness, and the use of toys in the car seats. The knowledge assessment was author-created based and questions were in multiple choice format and derived from important components of car seat safety as well the associated lecture. For example, the following was utilized assess caregiver knowledge of car seat harness tightness, “How tight should the hardness straps be?”: (a) Enough to pinch an inch, (b) Enough for two fingers underneath, or (c) closest to body. The same seven questions were used between the pretest and posttest to determine if the program impacted caregiver knowledge (Table 1). The assessment was available in English, Spanish, and Arabic, and non-English speakers had the program translated in real-time by a translator who also was available if questions rose during either the pre or posttests.

**Table 1:** Pre-Post knowledge assessment items. (* = Correct Responses)

Question	Choices
How long should your child remain in his/her car seat?	(1) Until 1 year of age, (2) Until 2 years of age, (3) Depends on height weight, (4) Until his/her feet touch the back of the front seat, or (5) As long as possible*
The appropriate car seat depends on what? Select all that apply	(1) Weight*, (2) Height*, (3) Model, (4) Car, and (5) Age
Until what age does your child have to remain in a car seat?	(1) 2 years, (2) 4 years, (3) 8 years*, (4) 13 years, or (5) Parental decision
Should coats be worn in a car seat?	(1) Yes, (2) No*
How tight should the harness straps be?	(1) Enough to pinch an inch*, (2) Enough for two fingers to slide underneath, or (3) As close as possible to the body
Should you let your child have toys in the car seat?	(1) Yes, (2) No*
How tight should the seat be in the car?	(1) Movable less than 1 in.*, (2) Movable less than 2 in., or (3) Tight against front seat

### Data analysis

Participants were assigned a randomized, numeric participant ID (e.g., 1, 2,,3…) to de-identify and link pre-post responses. Data from the paper surveys was entered in Microsoft Excel and scores were paired from pre to post. Responses to the questions were dichotomized into correct or incorrect responses. Participants total scores out of seven were summed on both the pre-test and post-test then a paired *t-*test was conducted to display differences between pre-posttest. Effect size (Cohen’s *d*) was calculated to identify the magnitude of difference. Performance was then analyzed by question, calculating the number of participants that correctly answered in on each question. Knowledge assessments by question were then compared within-subject using a paired chi-square McNemar’s test to determine differences from pre-posttest.

## Results

**Fig. 1:**
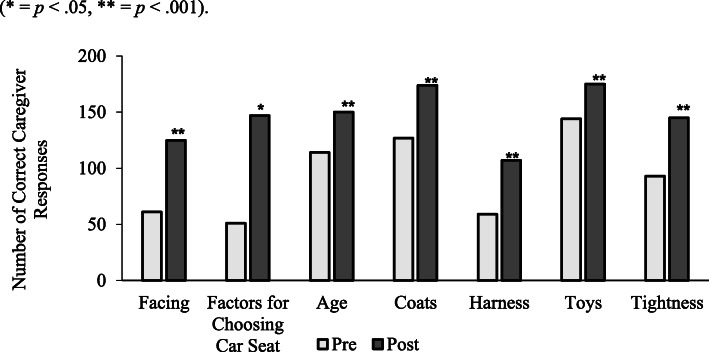
Number of subjects that answered each question correctly on the pre-test and post-test. (* = *p* < .05, ** = *p* < .001)

Caregivers (*N* = 200) in the present study displayed significant knowledge increases after the car seat course. On average, caregivers answered 46% of questions correctly before the course (*M* = 3.25, *SD* = 1.46), while the average on the posttest was 73% (*M* = 5.12, *SD* = 1.71): *t* (199) = − 12.56, *p* < .001, *d* = 1.17. Moreover, caregivers reported significant knowledge gains in all seven areas after the car seat program (*p* < .001) with the exception of choosing the appropriate car seat, which was significant, however, at the (*p* = .05) level. Figure 1 displays the correct number of responses, out of the sample, before and after the intervention. Lastly, in a single, open-ended item distributed at the conclusion of the course, 96% caregivers were able to recall Pennsylvania state requirements for proper height and weight requirements.

## Discussion

The goal of the present study was to measure the effectiveness of a car seat program in an urban, Level I Pediatric Trauma Center on caregiver car seat knowledge to promote child safety. Moreover, the objective of this study was to assess if a CPST-led training program could provide similar efficacy as noted in past outreach efforts in an underserved area (Food Research and Action Center 2020). Results displayed that a car seat training program led by a CPST significantly increased caregiver car seat knowledge. The change from pre-post average scores produced a large effect (*d* = 1.17) providing evidence that almost 90% of the caregivers at the post-test time point scored higher than their pre-test scores (Decina et al. 2016). Caregivers also increased significantly for all seven major knowledge areas that were included in the assessment (*p* < .05; Fig. 1).

Results from the current study extend outreach literature and found evidence to support the adoption of car seat programs in hospital settings to assist families who may not generally receive education. Specifically, Robitaille and colleagues (1990) attempted to provide car seats to low-income families to increase their use but noted that caregivers still did not possess knowledge to properly use them (Sullivan and Feinn 2012). Similarly, around the county, car seats are often distributed to parents at newborn discharge, but research has shown that even when installed by a CPST, parents are still uncomfortable using the seat (Robitaille et al. 1990). Therefore, the present study addressed gaps in both of these studies, by providing car seats, installing them for caregivers, and educating them on proper use to hopefully provide caregivers with the ability to install seats independently.

Providing car seat education and other outreach programs at hospitals eliminates barriers that low-income families may encounter seeking similar information elsewhere. Specifically, monetary costs of health care create significant disparities, especially in preventative medicine (Harper et al. 2015). However, including education as a part of routine hospital stays or visits could assist individuals who may not be able to access these programs in other areas and limit costs of separate trips (Hoffman et al. 2016). Additionally, including prevention programs can eliminate future injury and promotes holistic patient care (Steketee et al. 2017). Therefore, providing in-hospital access reduces cost and can prevent future life-threatening injuries from caregivers not properly using car seats.

Children who are not in car seats during an MVC are 71% more likely to suffer a fatal injury than those who are properly fitted and using car seats (The Centers for Disease Control and Prevention 2020). Given the importance of car seats for prevention and safety purposes, it is alarming that 56% of children aged 4–8 years have been found riding in cars unrestrained and that caregivers generally do not receive training on car seats in low-income areas (Sullivan and Feinn 2012; Horwitz et al. 2020; Apsler et al. 2002). Thus, the present study describes a program that was successful in increasing knowledge in caregivers who generally do not receive this type of training, which hopefully will decrease the broad disparity in rates of car seat usage.

Results of this hospital led program are encouraging, especially for other institutions located in generally underserved areas. Notably, the study included caregivers who had a relatively low baseline understanding (48%) of car seats and by providing them with 1 h of education, their knowledge significantly during the program (*p* < .001). In addition, this program provides initial evidence that car seat programs, even when short, can possibly provide life-saving information to caregivers to keep children safe in the event of an MVC.

### Limitations and future directions

The present study is not free of limitations. First, the current study is cross-sectional and only measures knowledge increases after one session. Therefore, true understanding beyond a short-term effect, cannot be assumed nor measured. Second, during survey design, the authors attempted to make the questions at an eighth-grade reading level; however, certain caregivers may not have comprehended or understood the items based on their reading level. The survey was initially designed by a group of outreach professionals; however, a strong limitation is the lack of previous use of such tool for data collection. While translators and amended surveys were available for non-English speakers, it is unclear if caregivers who received the program through translation found it as helpful. This could perhaps fuel future directions of car seat education research. Lastly, besides the hands-on component of training, caregiver ability to install a car seat or their comfort to do so was not measured. We did not measure the hands-on component, as we wanted to reserve this time to have the caregivers be able to ask questions to the CPST to assure proper fitting; however, future studies could nevertheless measure caregiver ability to install car seats properly after educational interventions.

Future studies should longitudinally explore the impact of car seat education to understand if programs have a long-lasting effect. This could be done by doing pre-post surveys over a lagged period of time (e.g., 6 months or a year between). Additionally, measuring caregiver comfort with installing car seats and making necessary adjustments without a CPST present would be important to understand to assist with the development of future programs. Also, the current study was limited by the data it was able to collect; however, future research should attempt to collect descriptive information about the sample including their child’s age, race, gender, and other demographic information. Further, attempting to provide education online via video and measuring the efficacy of a virtual program would be useful to avoid the burden of holding many sessions for CPSTs.

## Conclusions

Hospital-led car seat programs can increase safety and legal knowledge in low-income families by implementing a single, one-hour class. The present study found that using CPSTs and hospital resources may limit some pre-existing preventative healthcare barriers by providing caregivers education while they are at a hospital with their child receiving treatment. These findings should encourage other institutions to promote car seat education.

## Data Availability

Program materials can be requested and distributed through RB.

## Abbreviations

MVC: Motor Vehicle Collision
CDC: Centers for Disease Control and Prevention
SES: Socioeconomic Status
CPST: Child Passenger Safety Technician
